# Evidence for the Paleoethnobotany of the Neanderthal: A Review of the Literature

**DOI:** 10.1155/2016/8927654

**Published:** 2016-10-24

**Authors:** Gerhard P. Shipley, Kelly Kindscher

**Affiliations:** ^1^Indigenous Studies Department, University of Kansas, Lippincott Hall, 1410 Jayhawk Boulevard, Lawrence, KS 66045, USA; ^2^Kansas Biological Survey, University of Kansas, 2101 Constant Ave., Lawrence, KS 66047, USA

## Abstract

Our perception of our closest human relatives, the Neanderthals, has evolved in the last few decades from brutish ape-men to intelligent archaic human peoples. Our understanding and appreciation of their cultural sophistication has only recently extended to their diet. Only within the last few years, with new techniques and a shift in focus, have we begun to truly investigate and understand the role of plants in their diet and culture. The more we learn about Neanderthals, the more we realize that biological and cultural distinctions between them and us were relatively small. Given that we coexisted and likely interacted with them for thousands of years, the more we learn about them, the better we may understand our own past. In that light, we review the current evidence, derived from such sources as plant remains (e.g., starch, pollen, phytoliths, and seeds) in soil and dental calculus, dental and tool wear, coprolites, and genetics, for Neanderthal's nutritional, medicinal, and ritual use of plants, which includes 61 different taxa from 26 different plant families found at 17 different archaeological sites. Further, we updated and standardized botanical nomenclature from many sources published over many decades to provide a more stable foundation for future work.

## 1. Introduction

*Homo neanderthalensis* (alternatively,* H. sapiens neanderthalensis*) was a late archaic form of* H. sapiens* that diverged from modern human lineages no earlier than ~500k years ago [1] and had largely disappeared from Europe and Asia by ~41k–39k years ago [2], though evidence from Gibraltar suggests that some may have survived there until ~28k–24k years ago [3]. During that time, they established themselves in differing environments across the Middle East, Europe, and Asia and interacted with modern humans beginning between ~80k years ago [1] and ~52k years ago [4]. Neanderthals lived in a variety of environments, from the colder regions of Northern Germany and Siberia's Altai Mountains to the warmer regions of Mediterranean Gibraltar and the Levant. Over time, environmental changes likely impacted their access to food, and they clearly adapted in order to survive as long as they did. Along the way, Neanderthals developed complex tools (e.g., [5]), likely intentionally buried their dead (e.g., [6]), may have created cave art (e.g., [7]), very likely made ornamentation (e.g., [8]), likely used symbols and very likely had a spoken language [9], and mated with modern humans (e.g., [4]). However, despite what the dietary behaviors of our closest hominin relatives could potentially tell us about our own early history, we found no comprehensive review of the paleoethnobotany of the Neanderthal, and so we gleaned the literature for evidence of their nutritional, medicinal, and ritual uses of plants. Further, having collected this information from many sources published over many decades, we updated and standardized botanical nomenclature to provide a more stable foundation for future work.

## 2. Methods

We searched several hundred databases, including JSTOR, Academic OneFile, Anthropology Plus, AnthroSource, ScienceDirect, ProQuest, SAGE, SpringerLink, and Wiley Library Online, containing full-text articles from over 110,000 journals for relevant keywords and variants thereof (e.g., Neanderthal/Neandertal, paleoethnobotany/ethnobotany, paleoarchaeology/archaeology, plant, diet) to identify relevant sources of information. We also examined the references cited by these initially identified sources, and we returned to the online databases and searched for the names of the authors of all of the identified sources. We examined every reference cited herein except for the few that are in languages other than English, and for those we relied on the interpretations of others. Lastly, we updated and standardized the nomenclature for all plant identifications.

## 3. Results and Discussion

### 3.1. Methods for Determining Plant Contribution to Diet

The contribution of plants to Neanderthal diets is less well-studied and understood than the contribution of animals, partly because recovering plant remains can be difficult and often was not prioritized in past archaeological efforts. Furthermore, the “top predator” aspect of Neanderthal subsistence behavior was long overemphasized, and the contribution of plants to their diets was largely ignored [10]. In that regard, there may have been a degree of predetermination behind claims about Neanderthal diets. It was simplistically reasoned that modern humans living in cold environments eat more meat than plants; therefore, Neanderthals ate more meat than plants and the minor contribution of plants can be largely discounted. This ignores the fact that some Neanderthals lived in warmer Mediterranean and Middle Eastern environments and had access to a larger variety of plants (though, of course, increased access need not necessarily have resulted in increased use).

There may also have been some racial bias against Neanderthals, as reflected in characterizing the hunting of many different taxa by modern humans as “increased diet breadth” but by Neanderthals as “opportunistic” and, conversely, in characterizing the focus on a single prey species by modern humans as “specialization” but by Neanderthals as “an inability to exploit diverse resources” [11]. There may even have been some gender bias to the extent that hunting was long considered a masculine activity and therefore overvalued and overemphasized by predominately male researchers, while the gathering of plants was considered to be a feminine activity and therefore undervalued and underemphasized. More generally, Neanderthals have long been characterized by many researchers as cognitively inferior to modern humans, and, as a result, subtle differences have tended to be overinterpreted [5]. Whatever the reason, “our ideas of Neanderthal subsistence are biased by … a deep-seated intellectual emphasis on big game hunting” [12].

Plant remains are less likely to survive than animal remains, so “plant foods are almost invisible in the archaeological record” [13]. One technique for investigating the contribution of plants to Neanderthal diets is to infer their presence and use based on paleoecological reconstructions. If it can be shown that sites supported both Neanderthal and modern human populations and if it can be shown that Neanderthals' environments, behaviors, and biology were sufficiently similar to those of modern humans, then it may be possible to make valid analogies.

Micro- and macroscopic wear patterns and plant residues on Neanderthals' stone tools and even their teeth can provide evidence of plant use. Analyses of Neanderthals' teeth for dental micro- and macrowear have indicated that, like modern human foragers, Neanderthals' diets varied across environments at different latitudes. Dental microwear signatures are relatively dynamic depending on diet and may capture a few days to a few weeks of an individual's diet before death [14]. For example, Lalueza et al. [15] compared dental microwear patterns from modern human groups and Neanderthal groups from various sites, and found that Neanderthal patterns fell within or close to those of modern hunter-gatherers subsisting on a largely carnivorous diet, with a few exceptions indicating a more mixed diet. Fiorenza et al. [16] compared dental macrowear patterns from both groups and concluded that Neanderthals similarly tended toward more varied diets in warmer climates and more protein-based diets at higher latitudes. However, it should be noted that Pérez-Pérez et al. [17] found that such wear can be affected by differential dietary behaviors of individuals and by postmortem actions and therefore may not be a reliable indicator of general dietary behavior. Importantly, the use of tools on plant material is not definitive evidence of the consumption of that material, and even the chewing of plant material does not necessarily mean that the material was purposefully ingested.

Another technique is to examine Neanderthals' coprolites for undigested plant matter such as seeds, pollen, and phytoliths. Again, however, it is not necessarily the case that plant materials found on coprolites were consumed. Windborne pollen and other materials present in the environment may have been added after excretion. Also, phytoliths found in coprolites may have been consumed by herbivores whose digestive systems were then consumed by the depositors of the coprolites. Such predigested plant material, or chyme, was an important source of vitamins and minerals for modern humans in northern environments, and Neanderthals may have similarly consumed the stomachs and stomach contents of their prey [18, 19]. Sistiaga et al. [20] asserted to have identified 50,000-year-old coprolites as having been deposited by Neanderthals. However, coprolites of sufficient age are often so old that determining the species that deposited them or even confirming them as being coprolites may be impossible [21, 22].

Ratios of stable carbon (^13^C) and nitrogen (^15^N) isotopes in tissues such as collagen and tooth enamel can provide a record of an individual's diet over the last decade or so of life. More specifically, the ^13^C and ^15^N values from these tissues reflect the ^13^C and ^15^N values of dietary protein. Isotope analyses of Neanderthals from many sites [13, 23, 24] indicate a high protein, high trophic level diet similar to or even exceeding that of top predators such as wolves and hyenas. However, ^13^C and ^15^N analyses are only indicative of total protein consumption [24, 25]. Plants have less protein than meat, so plants are almost invisible in a mixed diet containing even a moderate amount of meat [10, 24]. Isotope analysis has been successfully performed on Iberian Neanderthals [26], but no collagen has been recovered from samples from Levantine sites where the consumption of plants may have been even greater [10].

One study attempted to analyze strontium (Sr)/calcium (Ca) and barium (Ba)/Ca ratios in Neanderthal bones from a site at Saint-Cesaire, France, and concluded that “the percentage of plants in the Neanderthal's diet must have been close to zero” [27]. However, this study examined only a single Neanderthal individual. Also, some plants and plant parts are known to be low in Sr, Ca, and Ba, and the study did not determine the ratios of these elements in all potentially edible species, including any plants that have bulbs, tubers, or corms [10].

An even less direct technique is to infer plant consumption based on genetic adaptations favoring plant digestion. For example, humans produce more salivary amylase than other higher primates, and salivary amylase breaks down starch molecules into accessible sugars [28]. As the main component of cereal grains, tubers, corms, bulbs, many nuts, and some inner bark, starch provides the most direct dietary source of glucose which is essential to producing metabolic energy [28]. An increase in genes that code for salivary amylase production may have facilitated a dietary shift to geophytes by early hominins [28], such as* Homo erectus*, and may have allowed them to rely more heavily on them [29], which may, in turn, have been a significant factor in their expansion into different environments [30]. Again, however, the ability to break down starch need not necessarily have resulted in the consumption of starchy plant materials by Neanderthals.

A recent technique is to examine dental calculus on Neanderthal teeth for plant material. Calculus is composed of mineralized plaque which generally accumulates over an individual's life and may therefore provide information about dietary behavior over a relatively long period [31]. Dental calculus adheres strongly to teeth and has been found on the teeth of a hominid dated to ~1.8 million years ago [32]. Most of the research on dental calculus has focused on extracting and identifying plant microfossils [33, 34]. Here again, plant material found in dental calculus may be the result of eating chyme, but a general lack of phytoliths suggests that starch grains found in dental calculus result from the primary consumption of plant material rather than consumption of chyme because most large herbivores consume large amounts of phytolith-rich grasses.

Despite their limitations, together these techniques have begun to produce an apparently valid and reliable view of the contribution of plants to Neanderthals' diets. As shown in Tables 1 and 2, these techniques have, to date, been used to discover 61 different taxa from 26 different plant families used by Neanderthals at 17 different archaeological sites.

### 3.2. Neanderthal Diet in General

The high proportion of very large herbivores in Neanderthals' diet indicates that they were the top predator in their environments [13, 24]. Based on analyses of animal remains [35], carbon and nitrogen isotopes, and energy requirement estimations [36], some studies have concluded that Neanderthal diets were more narrowly focused than those of modern humans on medium- and large-sized animals, with very little contribution from plants. For example, isotope analyses of Neanderthal remains from Marillac, France [37], Payre, France [38], Okladnikov Cave, Siberia [39], and sites along the central and southeastern Mediterranean coast of Iberia [26] have concluded that Neanderthals obtained most of their dietary protein from medium- and large-sized herbivores. Such studies generally admit that “these analyses cannot rule out plant consumption because of their methodological limitations” but still take the position that “Neanderthals did not regularly consume plant protein,” and while “there is evidence that some Neanderthal groups in certain areas … may have had a wider diet that included plants and smaller animals, this pattern is not very widespread” [26].

However, nutritional and energetic studies have indicated that Neanderthals could not have survived solely on terrestrial game [18], and plants must have provided carbohydrates and at least some of the required nutrients and calories [10, 26, 31]. The physiological limit on the amount of protein from lean meat requires that plant protein be part of the diet. At the very least, excess protein leads to a build-up of amino acids and ammonia which the consumption of plants can ameliorate [10, 40, 41]. More specifically, “lean meat can compose no more than 35% of dietary energy before a protein ceiling is reached,” and exceeding this threshold can have detrimental physiological effects [10]. For example, [18] assumed a 5,500 calories-per-day diet and concluded that such a diet derived exclusively from large, terrestrial herbivores “would kill a pregnant Neanderthal woman and her developing fetus … due to toxic levels of protein intake,” as well as toxic levels of vitamin A, niacin, iron, zinc, and selenium, and a severe under-consumption of carbohydrates, vitamin C, and calcium. Thus, “Neanderthals must have consumed greater amounts of nonterrestrial mammal foods than the archaeological record suggests” [18]. For example, El Zaatari et al. [42] examined dental microwear in twenty-five adult Neanderthals from nineteen western Eurasian sites and concluded that while their diets consisted predominately of meat, “plant foods did form an important part of the diet of at least some Neanderthal groups” and “the proportion of plant foods in the Neanderthal diet appears to have increased with the increase in tree cover.” Hockett and Haws [43] even hypothesized that because southern Neanderthals had greater access to plants, they likely incorporated greater amounts of essential nutrients into their diet than did northern Neanderthals and, therefore likely were, on average, healthier and lived longer than their northern counterparts.

Of course, “Neanderthals were certainly not vegetarians” and “it is far easier to discuss Neanderthals in terms of animal food consumption,” so we are only slowly moving “beyond the ‘and they also ate plants' stage of research” [10]. Studies that emphasized understanding plant consumption have found considerable complexity in Neanderthal foraging, including extensive use of a variety of plant materials when they were available, and these studies are “helping to correct the ‘meat fixation' of past subsistence studies” [44].

### 3.3. Current Evidence for the Consumption of Plants by Neanderthals

**Figure 1:**
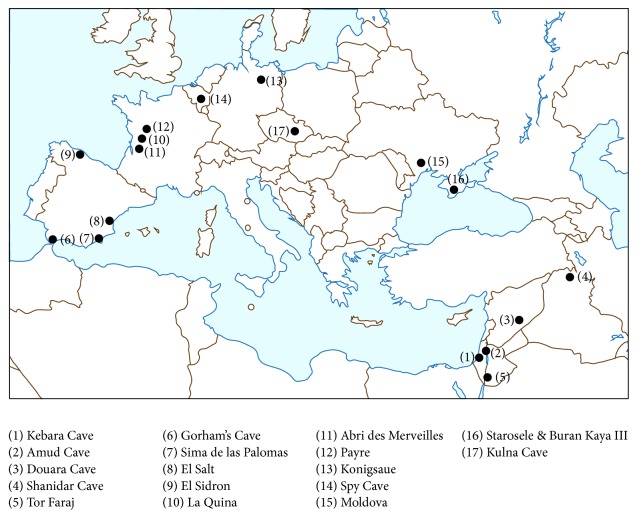
Locations of archaeological sites associated with evidence for plant use by Neanderthals.

Neanderthals likely consumed a mixed diet of animals and plants, including roots or bulbs during cooler periods [45]. Henry et al. [46] analyzed starch grains and phytoliths in dental calculus and on stone tools for several populations of Neanderthals and early modern humans in Europe, the Middle East, and Africa and found that Neanderthals across the entire range probably consumed as many plant species as did modern humans. Several plants identified from calculus require moderate to high levels of preparation (e.g., removal of the inedible husks of grass seeds) before consumption, and recent evidence for the cooking of plants, in the form of smoke-related compounds, methylated lipids, and heat-cracked starch grains trapped in calculus, indicates a previously unrecognized level of sophistication in the Neanderthal diet [33, 34]. As seen in Figure 1 and Tables 1 and 2, a number of studies have found evidence for the nutritional, medicinal, and ritual use of plants by Neanderthals at sites throughout the Middle East and Europe. We have updated the identifications of these plants to reflect the latest nomenclature.

In the Middle East, charred seeds and nuts were recovered from a Neanderthal site at Kebara Cave, Israel, including the carbonized seeds of wild peas [47–49]. Most of these were identified as belonging to forty-eight different taxa, primarily legumes (Fabaceae) [47]. Seeds from three taxa came from edible geophytes: wild radish (*Raphanus raphanistrum* L.), nut-grass (*Cyperus* sp.), and bulbous barley (*Hordeum spontaneum* K. Koch) [10]. These Neanderthals were likely consuming a significant amount of legumes and a smaller amount of grass seeds (*Poaceae*), including barley grains, and acorns, pistachios, and fruits may also have constituted a seasonally significant part of the diet, but there is little evidence for the consumption of roots or cereals [47, 50]. Albert et al. [51] concluded that the majority of plant material brought into the cave was used as fuel but further concluded that plant materials other than those used as or clinging to fuel were also brought into the cave, including yellowspine thistle (*Cirsium ochrocentrum* A. Gray) and fennel. The presence of wild peas suggests that Kebara Cave was occupied during the spring because wild peas are available in the area in April and May [48, 50].

Similarly, Madella et al. [52] examined phytoliths from Amud Cave, Israel, and concluded that Neanderthals used plant materials extensively. The presence of palm (Palmae) and fig-tree family (Moraceae) phytoliths indicates that they may have consumed palm fruits and figs, and other phytoliths indicate that they may have gathered wild cereals and other edible grasses [52]. However, Albert et al. [53] examined Tabun Cave, Israel, and found phytoliths derived almost entirely from wood and bark used as fuel and not from grasses or leaves.

Akazawa [54] reviewed carbonized plant remains from a Neanderthal site at Douara Cave, Syria, including nettle tree (*Celtis australis* L. and* C. tournefortii* Lam.) endocarps and Boraginaceae nutlets and seed spodograms, as well as small numbers of* Pinus*,* Juniperus*,* Artemisia*, Chenopodiaceae, Poaceae, and* Pistacia* pollen grains. Henry et al. [55, 56] examined a site at Tor Faraj, in Jordan, and found monocot phytoliths from the* Pooid* grass subfamily, rush* Cyperus*, and the reed grass,* Phragmites*. Furthermore, “the distributions of phytoliths of date palm (*Phoenix dactylifera* L.), seed husks, grasses (monocots), and woody plants (dicots), along with starch grains, provide important evidence of plant-related activities in the shelter” [56]. The starch grains are likely derived from pistachios, other nuts, roots, and tubers.

Henry et al. [34] found that dental calculus from Shanidar Cave, Iraq, contained starches from Triticeae grass seeds, a probable legume starch (subfamily Faboideae and possibly tribe Fabeae), a starch from an edible geophyte, phytoliths from date palms (*Phoenix* spp.), and several other probable tree fruit phytoliths. The Triticeae starches derived from wild relatives of barley (*Hordeum* spp.) and several others exhibited clear evidence of having been cooked [34]. Stone tools from the same site yielded Triticeae starches, date palm phytoliths, and a variety of other starch types, though none of these appeared to have been cooked [46].

In Europe, Barton et al. [57] recovered charred remains of edible plants from a Neanderthal site at Gorham's Cave, Gibraltar, including wild olives (*Olea* sp.) and stone pine nut (*Pinus pinea* L.). Beach cobbles found in the cave among charred nut shells and other organic remains exhibited percussion marks and may have been used in plant processing [57]. Soil layers from the same site yielded Triticeae and Andropogoneae or Paniceae grass seed starches and two other unidentified types, though one is likely from a USO [46]. Salazar-García et al. [26] studied dental calculus and stone tools from Sima de las Palomas, Spain, and found microfossils indicating that Neanderthals consumed “a diversity of plant types, such as leafy matter indicated by polyhedral phytolith multicells, hard endosperm of seeds or nuts as well as grass seeds and possibly underground storage organs.” Furthermore, these Neanderthals suffered from a high number of dental caries, which suggests increased carbohydrates in their diet [26]. Sistiaga et al. [20] analyzed coprolites from a site in El Salt, Spain, and based on the presence of phytosterol metabolites concluded that these Neanderthals consumed plants as well as meat. Hardy et al. [33] examined dental calculus from five Neanderthals from a site at El Sidron, Spain, and found “chemical evidence consistent with wood-fire smoke, a range of cooked starchy foods, and two plants known today for their medicinal qualities,” yarrow and chamomile, which are discussed in the next section. In particular, certain starches suggested nontuber plants, certain phytoliths suggested grasses (Poaceae), certain acids suggested nuts, pyrolyzed lutein (a xanthophyll) suggested green vegetables, starches of different shapes and sizes suggested more than one plant genus, and edge-cracking of certain starch granules suggested they were cooked [33].

Hardy [58] found plant residue on stone tools from a Neanderthal site at La Quina, France, which indicated that plants were processed. Henry et al. [46] studied stone tools from the same site and found a large number and variety of plant microremains, including several from the* Triticeae* tribe, and several were damaged in a manner that suggested processing. Stone tools from Abri des Merveilles, France, yielded two different kinds of Triticeae starches, some likely grass starches from the Andropogoneae or Paniceae tribes, and some unidentified grains [46]. Hardy and Moncel [12] examined stone artifacts from Payre, France, and found plant residue in the form of starch grains, plant tissue, raphides, phytoliths, wood, and resin.

Grünberg [59] found evidence for the manufacture of birch-bark pitch at a Neanderthal site at Konigsaue, Germany, which would likely have been used to affix stone points or blades to hafts of wood, antler, or bone. Sandgathe and Hayden [60] suggested that Neanderthal artifacts recovered from European (primarily German) sites “may have been bark peelers used to procure inner bark from trees and that this was an early and widespread Paleolithic activity” (but note that the authors acknowledged they could be interpreted as digging sticks, and Thieme [61] interpreted them as throwing sticks). Typically, edible inner bark consists of the immature secondary phloem and vascular cambium which is harvested in the spring and early summer while it is still soft and moist and before it differentiates into tougher tissues. The great apes eat inner bark, and “ethnographic evidence indicates that inner bark was exploited as a food resource all across the temperate globe” by modern Indigenous peoples [60], and therefore Watanabe [62] suggested that the Neanderthals of Europe likely also ate inner bark.

Henry et al. [46] found that dental calculus from Spy Cave, Belgium, contained a number of starches of unknown types. However, one-third were from a single type of water lily rhizome (*Nymphaea*) family, and some of the starches were similar to grass seeds in the Andropogoneae or Paniceae tribes [46]. Mazza et al. [63] found evidence for birch-bark pitch used as a mastic in the hafting of stone tools found among elephant and rodent remains in deposits in central Italy. Hardy [10] reported that both Chernysh [64, 65] and Paunescu [66, 67] found possible grinding stones with use-wear evidence indicating plant processing at the Neanderthal sites of Moldova I and V, along the Dniester River in eastern Europe. Hardy et al. [68] conducted a mixed residue and use-wear analysis of stone tools from Neanderthal sites at Starosele and Buran Kaya III, Crimea, and found starch grains and plant remains and concluded that starchy plant storage organs had been used as part of the binding for a tool handle or as food. Henry et al. [46] found that one-quarter of the starches from dental calculus from Kulna Cave, Moravia, were from grass seeds in the Triticeae tribe, which includes the wild relatives of wheat and barley.

More generally, Hardy [10] made a compelling circumstantial argument for the consumption of geophytes by European Neanderthals based on the geographic and seasonal availability and nutritional content of various exemplary geophytes. Given their characteristics, “it would be surprising if [geophytes] were not part of the Neanderthal diet” [10]. Henry et al. [46] have also asserted that Neanderthals in Europe and the Middle East consumed geophytes and grass seeds.

### 3.4. Current Evidence for the Use of Plants by Neanderthals in Medicine and Ritual

Many animals have been observed to practice some degree of self-medication using plants, whether for their prophylactic or therapeutic effects. This may result from natural selection for a predisposition to seek out and use plant tissues with particular markers [69]. Specifically, the medicinal properties of some plants result from secondary metabolites that are toxic to bacterial pathogens, parasites, or insects that attack the plants, and a bitter, astringent, or otherwise repellant taste which signals the unpalatability of a plant part is a common indicator of these compounds [69]. Among hominids, the use of medicinal plants is also perpetuated through observation and social learning that specific plant parts, often prepared in specific ways, are at least somewhat effective at treating specific illnesses [69].

Neanderthals “had a sophisticated knowledge of their natural surroundings, and were able to recognize both the nutritional and the medicinal value of certain plants” [33] and therefore almost certainly also used them. Hardy et al. [33] examined the dental calculus of Neanderthals from El Sidron, Spain, and found compounds from yarrow and chamomile, both of which have medicinal properties, and both of which are bitter tasting and were therefore likely chosen and ingested for their medicinal properties rather than consumed as food [41]. In fact, Lalueza-Fox et al. [70] found that Neanderthals from the El Sidron site had the TAS2R38 gene for perceiving the taste of bitterness and argued that the presence of this gene suggests a predisposition to eating plants because it allows for perceiving and thereby generally avoiding potentially toxic compounds. In that light, Hardy et al. [33] claimed to “offer the first evidence for the use of medicinal plants by a Neanderthal individual.” However, Krief et al. [71] noted that, in addition to self-medication, there were other possible explanations for the use of these plants, including flavoring food, reducing the risk of infection by bacteria or parasites in the digestive tracts of prey (to which Neanderthals may have been exposed by eating chyme), and medicating (as opposed to* self*-medicating).

Perhaps the most intriguing potential example of Neanderthals and the medicinal and/or ritual use of plants is that of a grave at Shanidar, Iraq, in which a Neanderthal was buried with several plants recognized as having medicinal properties, some of which were flowering when placed in the grave [72–74]. Solecki [72] identified the plants as belonging to the family Compositae, genus* Achillea* (yarrow), genus* Centaurea* (which includes cornflower and St. Barnaby's thistle), and genus* Senecio* (ragwort); the family Liliaceae, genus* Muscari* (grape hyacinth); the family Gnetaceae, genus* Ephedra* (joint pine); and the family Malvaceae, genus* Althaea* (hollyhock). The clustering and arrangement of many pollen grains indicates that full flowers were placed into the grave, and based on when these flowers bloom, “one may assume that the placement of the Neanderthal man … on a bed of flowers occurred more than 50,000 years ago between the end of May and the beginning of July” [73]. Solecki [72] went so far as to speculate that this Neanderthal “was not only a very important man, a leader, but also may have been a kind of medicine man or shaman in his group.” However, Sommer [75] argued that a small gerbil-like rodent native to the region could have introduced enough flower heads into the cave to account for the pollen found in the grave, and the debate over whether the Shanidar burial provides evidence of the medicinal or ritual use of plants is ongoing. For example, Nadel et al. [76] asserted that this evidence of rodent activity “cast serious doubts” on the interpretation that the flowers were placed by Neanderthals, but Guerra-Doce [77] acknowledged only that some scholars “dispute the idea that these plants were the result of a ritual deposition.”

“It is extremely likely that, as practicing naturalists (and early-day ecologists?), the Neanderthals must have known and appreciated all of their environment, since their very existence depended on it” [72]. After all, “the care that is needed in the selection and ingestion of plants so as to exclude noxious secondary compounds is essential for survival and requires methods of knowledge transfer” [41]. Both good and bad experiences with various plants would have been passed down [72] as part of Neanderthal ecological knowledge. Thus, Neanderthals “had a sophisticated knowledge of their natural surroundings, and were able to recognize both the nutritional and the medicinal value of certain plants” [33].

### 3.5. Limitations

This review of evidence for the ethnobotany of the Neanderthal is necessarily limited in many ways. For example, we have not associated dates with any of the evidence. In some cases, such as where evidence was found in distinct geological strata, estimated dates are generally available. However, this information quickly becomes complex, especially where evidence was found at different levels at the same site. Also, we have not discussed evidence of related plant use by modern humans. Some sites hosted both Neanderthals and modern humans at different times, and some evidence for similar uses of the same or similar plants may exist. There are many such interesting and important tangents that could be pursued, but they are beyond the scope of this paper.

## 4. Conclusion

Our understanding and appreciation of the cultural sophistication of Neanderthals are only now extending to their diet. Three decades ago we thought they were obligate scavengers, and two decades ago we accepted that they were highly effective hunters and top predators in their environments, but it has only been within the last decade or so that we have truly begun to realize the significance of plants in their diets and, perhaps more importantly, in their medicinal and ritual traditions. This advance is due partly to a paradigm shift away from the caveman caricature and partly to an increased emphasis on identifying evidence of plant use. Our review of the current evidence for dietary, medicinal, and ritual use of plants by these archaic human peoples reveals a promising trend toward a better understanding of them, which we believe will ultimately lead to a better understanding of ourselves.

## Figures and Tables

**Table 1:** Archaeological contexts and forms of evidence for plant use by Neanderthals at various locations.

Location	Archaeological context	Form of evidence	Citation
Abri des Merveilles, France	Stone tools	Starch	Henry et al. [46]
Amud Cave, Israel	Soil	Panicles, phytoliths	Madella et al. [52]
Douara Cave, Syria	Soil	Endocarps, seeds, spodograms, nutlets	Akazawa [54]
	Soil	Pollen	Akazawa [54]
El Salt, Spain	Coprolites	Phytosterol metabolites	Sistiaga et al. [20]
El Sidron, Spain	Dental calculus	Phytoliths	Hardy et al. [33]
	Dental calculus	Chemical compounds	Hardy et al. [33]
	Dental calculus	Starches, acids, pyrolyzed lutein	Hardy et al. [33]
	Genetics	TAS2R38 gene	Lalueza-Fox et al. [70]
Europe (primarily Germany)	Artifact	Wooden tool	Sandgathe and Hayden [60]
Gorham's Cave, Gibraltar	Artifact	Beach cobbles	Henry et al. [46]
	Soil	Plant remains	Barton et al. [57]
	Soil	Starch	Henry et al. [46]
Italy (central)	Stone tools	Pitch	Mazza et al. [63]
Kebara Cave, Israel	Soil	Acorns, nuts	Lev and Kislev [50]; Lev et al. [47]
	Soil	Phytoliths	Albert et al. [51]
	Soil	Seeds	Bar-Yosef et al. [48]; Lev and Kislev [50]; Lev et al. [47]; Hardy [10]
Konigsaue, Germany	Soil	Pitch	Grünberg [59]
Kulna Cave, Moravia	Dental calculus	Starches	Henry et al. [46]
La Quina, France	Stone tools	Large variety of microremains	Hardy [58]; Henry et al. [46]
Moldova I and V, Dniester River	Grinding stones	Use-wear	Chernysh [64, 65]; Paunescu [66, 67]
Payre, France	Stone tools	Plant residue including starch grains, plant tissue, raphides, phytoliths, and resin	Hardy and Moncel [12]
Shanidar Cave, Iraq	Dental calculus	Phytoliths, starches	Henry et al. [34]; Henry et al. [46]
	Soil	Starch	Solecki [72]
	Soil	Pollen	Solecki [72]
Sima de las Palomas, Spain	Dental calculus	Phytoliths, endosperm, dental caries	Salazar-García et al. [26]
Spy Cave, Belgium	Dental calculus	Starches	Henry et al. [46]
Starosele and Buran Kaya III, Crimea	Stone tools	Starches and other plant remains	Hardy et al. [68]
Tor Faraj, Jordan	Soil	Phytoliths, seed husks	Henry et al. [55, 56]

**Table 2:** Ethnobotanical remains recovered from Neanderthal archaeological sites, including 26 families and 61 unique species. Taxonomic identifications have been updated to reflect the latest nomenclature. Note: “cf.” denotes an unconfirmed identification.

Family	Genus, species & authority	Possible ethnobotanical use	Location	Citation
Anacardiaceae	*Pistacia atlantica* Desf.	Food—nuts	Kebara Cave, Israel	Lev et al. [47]
Anacardiaceae	*Pistacia *sp.	Food—nuts	Douara Cave, Syria	Akazawa [54]
Anacardiaceae	*Pistacia *sp.	Food—nuts	Tor Faraj, Jordan	Henry et al. [55, 56]
Anacardiaceae	*Pistacia vera *L.	Food—nuts	Kebara Cave, Israel	Lev and Kislev [50]; Lev et al. [47]
Apiaceae	*Foeniculum vulgare *Mill.	Food—leaves; medicine	Kebara Cave, Israel	Albert et al. [51]
Arecaceae	*Phoenix dactylifera *L.	Food	Tor Faraj, Jordan	Henry et al. [55, 56]
Arecaceae	*Phoenix* sp.	Food	Shanidar Cave, Iraq	Henry et al. [34]; Henry et al. [46]
Asteraceae	*Achillea millefolium* L.	Medicine	El Sidron, Spain	Hardy et al. [33]
Asteraceae	*Achillea *sp.	Medicine	Shanidar Cave, Iraq	Solecki [72]
Asteraceae	*Artemisisa *sp.	Medicine—nutlets	Douara Cave, Syria	Akazawa [54]
Asteraceae	*Carthamus* sp.	Medicine; dye plant	Kebara Cave, Israel	Lev et al. [47]
Asteraceae	*Carthamus tenuis *(Boiss. & Blanche) Borum	Medicine; dye plant	Kebara Cave, Israel	Lev et al. [47]
Asteraceae	*Centaurea *sp.	Possibly medicine/ritual	Shanidar Cave, Iraq	Solecki [72]
Asteraceae	*Cirsium ochrocentrum *A. Gray	Food—seeds; medicine	Kebara Cave, Israel	Albert et al. [51]
Asteraceae	*Matricaria chamomilla *L.	Medicine	El Sidron, Spain	Hardy et al. [33]
Asteraceae	*Senecio *sp.	Possibly medicine/ritual	Shanidar Cave, Iraq	Solecki [72]
Betulaceae	*Betula *sp.	Medicine/utilitarian	Konigsaue, Germany	Grünberg [59]
Betulaceae	*Betula *sp.	Medicine/utilitarian	Central Italy	Mazza et al. [63]
Boraginaceae	*Echium angustifolium/judaeum*	Food	Kebara Cave, Israel	Lev et al. [47]
Boraginaceae	*Onosma gigantea *Lam.	Unknown	Kebara Cave, Israel	Lev et al. [47]
Boraginaceae	*Onosma orientalis *(L.) L.	Unknown	Kebara Cave, Israel	Lev et al. [47]
Brassicaceae	*Raphanus raphanistrum *L.	Food—roots, herb—medicine	Kebara Cave, Israel	Hardy [10]; Lev et al. [47]
Caryophyllaceae	cf. *Silene aegyptiaca *(L.) L. f.	Medicine	Kebara Cave, Israel	Lev et al. [47]
Chenopodiaceae	*Chenopodium murale *L.	Medicine	Kebara Cave, Israel	Lev et al. [47]
Chenopodiaceae		Food	Douara Cave, Syria	Akazawa [54]
Cupressaceae	*Juniperus *sp.	Food/medicine	Douara Cave, Syria	Akazawa [54]
Cyperaceae	cf. *Cyperus*	Food—tubers	Kebara Cave, Israel	Lev et al. [47]
Cyperaceae	*Cyperus *sp.	Food—tubers	Kebara Cave, Israel	Hardy [10]
Cyperaceae	*Cyperus *sp.	Food—tubers	Tor Faraj, Jordan	Henry et al. [55, 56]
Equisetaceae	*Ephedra* sp.	Possibly medicine/ritual	Shanidar Cave, Iraq	Solecki [72]
Euphorbiaceae	cf. *Euphorbia aleppica *L.	Unknown	Kebara Cave, Israel	Lev et al. [47]
Euphorbiaceae	*Mercurialis annua *L.	Medicine	Kebara Cave, Israel	Lev et al. [47]
Fabaceae	*Astragalus echinus *L.	Food	Kebara Cave, Israel	Lev et al. [47]
Fabaceae	cf. *Scorpiurus muricatus *L.	Food	Kebara Cave, Israel	Lev et al. [47]
Fabaceae	cf. *Vicia narbonensis *L.	Food	Kebara Cave, Israel	Lev et al. [47]
Fabaceae	*Cicer pinnatifidum *Jaub.	Food	Kebara Cave, Israel	Lev et al. [47]
Fabaceae	*Hymenocarpos circinnatus *(L.) Savi	Unknown	Kebara Cave, Israel	Lev et al. [47]
Fabaceae	*Lathyrus hierosolymitanus *Boiss.	Food	Kebara Cave, Israel	Lev et al. [47]
Fabaceae	*Lathyrus inconspicuous *L.	Food	Kebara Cave, Israel	Lev et al. [47]
Fabaceae	*Lathyrus *sect*. Cicercula*	Food	Kebara Cave, Israel	Lev et al. [47]
Fabaceae	*Lens* sp.	Food	Kebara Cave, Israel	Lev et al. [47]
Fabaceae	*Pisum fulvum/Vicia narbonensis/peregrina*	Food	Kebara Cave, Israel	Lev et al. [47]
Fabaceae	*Pisum fulvum/Vicia palaestina*	Food	Kebara Cave, Israel	Lev et al. [47]
Fabaceae	*Scorpiurus muricatus *L.	Food	Kebara Cave, Israel	Lev et al. [47]
Fabaceae	*Trifolium* sp.	Food	Kebara Cave, Israel	Lev et al. [47]
Fabaceae	*Vicia cuspidata/lathyroides*	Food	Kebara Cave, Israel	Lev et al. [47]
Fabaceae	*Vicia ervilia *(L.) Willd.	Food	Kebara Cave, Israel	Lev et al. [47]
Fabaceae	*Vicia laxiflora/tetrasperma*	Food	Kebara Cave, Israel	Lev et al. [47]
Fabaceae	*Vicia lutea/sativa/sericocarpa*	Food	Kebara Cave, Israel	Lev et al. [47]
Fabaceae	*Vicia palaestina *Boiss.	Food	Kebara Cave, Israel	Lev et al. [47]
Fabaceae	*Vicia palaestina/sativa*	Food	Kebara Cave, Israel	Lev et al. [47]
Fabaceae	*Vicia palaestina/villosa*	Food	Kebara Cave, Israel	Lev et al. [47]
Fabaceae	*Vicia peregrina *L.	Food	Kebara Cave, Israel	Lev et al. [47]
Fabaceae	*Vicia pubescens *(DC.) Link	Food	Kebara Cave, Israel	Lev et al. [47]
Fabaceae		Food—wild peas	Kebara Cave, Israel	Bar-Yosef et al. [48]; Lev and Kislev [50]; Lev et al. [47]
Fabaceae		Food	Shanidar Cave, Iraq	Henry et al. [34]; Henry et al. [46]
Fagaceae	*Quercus *sp.	Food—acorns	Kebara Cave, Israel	Lev and Kislev [50]; Lev et al. [47]
Liliaceae	*Bellevalia *sp.	Food—bulb	Kebara Cave, Israel	Lev et al. [47]
Liliaceae	*Muscari* sp.	Possibly medicine/ritual	Shanidar Cave, Iraq	Solecki [72]
Malvaceae	*Althaea* sp.	Medicine	Shanidar Cave, Iraq	Solecki [72]
Malvaceae	*Malva* sp.	Medicine	Kebara Cave, Israel	Lev et al. [47]
Moraceae	*Ficus carica *L.	Food—fig fruits	Amud Cave, Israel	Madella et al. [52]
Nymphaea		Food—tubers, seeds	Spy Cave, Belgium	Henry et al. [46]
Oleaceae	*Olea *sp.	Food	Gorham's Cave, Gibraltar	Barton et al. [57]
Palmae		Food—palm greens and fruit	Amud Cave, Israel	Madella et al. [52]
Pinaceae	*Pinus pinea *L.	Food	Gorham's Cave, Gibraltar	Barton et al. [57]
Pinaceae	*Pinus *sp.	Food/medicine	Douara Cave, Syria	Akazawa [54]
Poaceae	*Aegilops geniculata/peregrina*	Food—grain	Kebara Cave, Israel	Lev et al. [47]
Poaceae	*Avena barbata/wiestii*	Food—grain	Kebara Cave, Israel	Lev et al. [47]
Poaceae	cf. *Brachypodium distachyon *(L.) P. Beauv.	Food—grain	Kebara Cave, Israel	Lev et al. [47]
Poaceae	cf. *Bromus*	Food—grain	Kebara Cave, Israel	Lev et al. [47]
Poaceae	cf.* Cynodon dactylon *(L.) Pers.	Food—grain	Kebara Cave, Israel	Lev et al. [47]
Poaceae	*Hordeum spontaneum *K. Koch	Food—grain	Kebara Cave, Israel	Hardy [10]; Lev and Kislev [50]; Lev et al. [47]
Poaceae	*Hordeum spontaneum/bulbosum*	Food—corms or grain	Kebara Cave, Israel	Lev et al. [47]
Poaceae	*Hordeum *spp.	Food	Shanidar Cave, Iraq	Henry et al. [34]; Henry et al. [46]
Poaceae	*Phragmites *sp.	Food/other	Tor Faraj, Jordan	Henry et al. [55, 56]
Poaceae		Food—grain	Kebara Cave, Israel	Lev and Kislev [50]; Lev et al. [47]
Poaceae		Food—grain	Amud Cave, Israel	Madella et al. [52]
Poaceae		Food—grass seeds	Douara Cave, Syria	Akazawa [54]
Poaceae		Food from multiple species	Gorham's Cave, Gibraltar	Henry et al. [46]
Poaceae		Food	El Sidron, Spain	Hardy et al. [33]
Poaceae		Food	La Quina, France	Hardy [58]; Henry et al. [46]
Poaceae		Food from multiple species	Abri des Merveilles, France	Henry et al. [46]
Poaceae		Food	Kulna Cave, Moravia	Henry et al. [46]
Poaceae	*Triticeae*	Food	Shanidar Cave, Iraq	Solecki [72]
Rubiaceae	*Galium* sect. *Kolgyda*		Kebara Cave, Israel	Lev et al. [47]
Ulmaceae	*Celtis australis *L.	Food—endocarps and seeds	Douara Cave, Syria	Akazawa [54]
Ulmaceae	*Celtis tournefortii *Lam.	Food—endocarps and seeds	Douara Cave, Syria	Akazawa [54]
Vitaceae	*Vitis vinifera *ssp. *sylvestris*		Kebara Cave, Israel	Lev et al. [47]
